# How artificial intelligence is shaping neuropsychology: A focus on cognitive assessment of neurodegenerative disorders

**DOI:** 10.1111/jnp.70009

**Published:** 2025-08-09

**Authors:** Michele Scandola, Maria Esposito, Riccardo Guidotti, Daniele Romano

**Affiliations:** ^1^ Department of Human Sciences University of Verona Verona Italy; ^2^ Psychology Department University of Milan‐Bicocca Milan Italy

**Keywords:** alzheimer, classification, machine learning, mild cognitive impairment, neurodegenerative disorders, parkinson, primary progressive aphasia

## Abstract

Artificial intelligence (AI) and machine learning (ML) algorithms are revolutionising the world, and they have the potential to revolutionise neuropsychology as well. A particularly fruitful field for this revolution is the cognitive assessment of neurodegenerative disorders, such as Alzheimer's disease, Parkinson's disease, Mild Cognitive Impairment and Primary Progressive Aphasia. This narrative review explores the impact of ML and AI in classifying these patients by using biomarkers or neuropsychological tests, using vast amounts of data and providing previously unattainable insights. Additionally, the article will evaluate the accuracies of several ML algorithms, such as support vector machines, random forest or convolutional neural networks. The article will also discuss the challenges related to ML like the risk of overfitting and the need for ML algorithms to execute a differential analysis among several pathologies—a capability that current research has yet to achieve fully. Furthermore, it proposes new directions to improve the clinical utility and accuracy of ML classification algorithms in neuropsychology, underlining the possibility for theoretical advancements based on the results of these classifications.

## INTRODUCTION

Advanced computational tools such as machine learning (ML) and artificial intelligence (AI) are increasingly shaping our daily lives. Their emergence has opened new directions and opportunities that, just a few years ago, might have been dismissed as the stuff of science fiction. Today, modern technologies can gather vast amounts of data, process it and generate responses using sophisticated algorithms. The pace of their evolution appears rapid and shows no signs of slowing.

Much has changed since the pioneering work of McCulloch and Pitts (1943), who conceptualised machines capable of emulating human cognitive processes. Like any transformative technology, the rise of AI has evoked a spectrum of emotions, from surprise and anxiety to eventual integration into daily life (Orben, 2020).

Despite concerns about the increasing digitalisation of our lives, the positive impact of these technologies is undeniable. For instance, AI‐driven diagnostic tools analyse medical imaging with remarkable accuracy, aiding in the early detection of diseases like cancer (Huang et al., 2020). Big data supports personalised medicine by analysing genetic information and patient histories to tailor treatments (Cirillo & Valencia, 2019). Furthermore, it is instrumental in tracking climate change by analysing global patterns such as temperature fluctuations and deforestation rates (Papadopoulos & Balta, 2022) while also optimising resource use in agriculture to promote sustainability (Javaid et al., 2023). Additionally, AI‐powered tools like real‐time speech recognition, language translation (Patil et al., 2020) and assistive technologies enhance accessibility for individuals with disabilities, fostering inclusivity (Zdravkova, 2022).

These advances also hold significant potential in clinical neuropsychology. Big data analysis, AI‐driven tools and other digital technologies are poised to assist clinicians in domains such as assessment, hypothesis generation and rehabilitation.

In assessment, these tools can analyse biomarkers such as vocal characteristics, eye‐tracking metrics and more nuanced electrophysiological signals like EEG patterns or neuroimaging data. They can also interpret the results of neuropsychological tests, providing deeper insights and identifying patterns that may not be immediately apparent. Additionally, AI systems like Large Language Models (LLMs) can engage in collaborative dialogues akin to Socratic maieutics, serving as tools to refine clinical hypotheses and better understand patients' conditions.

Finally, applying these tools can help make critical theoretical advances thanks to new classifications of variants of neuropsychological syndromes resulting from data‐driven approaches.

In particular, these technologies have been applied to neurodegenerative disorders. Neurodegenerative disorder is a broad classification comprising the most well‐known forms of dementia, such as Alzheimer's disease (AD), which degenerates the entorhinal and other memory‐related cortices, causing memory and other cognitive impairments (Dickerson et al., 2009); Parkinson's disease (PD), primarily affecting the substantia nigra, causing striatal dopamine deficiency with main clinical characteristics in the motor domain, such as bradykinesia and many non‐motor symptoms (Poewe et al., 2017); mild cognitive impairment (MCI), a class of neurodegenerative disorders that are characterised by cognitive decline greater than expected for an individual's age and education level, but that does not interfere notably with activities of daily life (Gauthier et al., 2006) and primary progressive aphasia (PPA), which is focal dementia characterised by an isolated and gradual dissolution of language function (Mesulam, 2001).

### Aim of the paper

This article aims to provide a narrative review and position paper on the current and emerging applications of AI—particularly ML—in clinical neuropsychology. While AI broadly encompasses a range of computational approaches designed to simulate human intelligence, our focus is specifically on ML techniques that are trained on neuropsychological test data to support diagnosis, classification and monitoring of cognitive impairment, with a particular emphasis on neurodegenerative disorders such as Alzheimer's disease, Parkinson's disease and related conditions. The manuscript discusses how ML can enhance cognitive assessment by increasing objectivity, identifying informative test features and potentially streamlining diagnostic protocols, but also how it can enhance neuropsychology from a theoretical point of view. In doing so, we critically examine both the benefits and limitations of these technologies, including challenges related to generalisability, overfitting and ethical use. Importantly, this article does not focus on other AI applications such as LLMs, chatbots or conversational AI systems, which, although relevant to the broader field of digital health, fall outside the scope of this review. Instead, our goal is to outline how data‐driven models based on structured neuropsychological data may shape the future of clinical neuropsychology and contribute to both scientific understanding and clinical care. Moreover, the manuscript includes a Glossary in Supplementary Materials to introduce and define most of the technical terms.

## MACHINE LEARNING ALGORITHMS

Classification algorithms are instrumental in the clinical context among the many ML algorithms.

In particular, support vector machines (SVMs), recurrent neural networks (RNNs), convolutional neural networks (CNNs), Multilayer Perceptron (MLP), k‐Nearest Neighbours (KNN), Naïve Bayes (NB), Deep‐Neural Network (DNNs), Gaussian Mixture Models (GMMs) and ensemble methods, such as Random Forest (RF), are the methods most commonly used for these purposes, thanks to their ability to classify complex clinical data with high accuracy and reliability (Saturi, 2023; Desai & Shah, 2021; Hu et al., 2016, see Table 1).

**TABLE 1:** Main strengths and weaknesses of different ML algorithms.

Algorithm	Strength(s)	Weakness(es)	Summary	Use case(s)	References
Logistic Regression	Interpretability, low computational costs, allows for regularisation (L1/L2) thus improving robustness	Assumptions on independence of errors, weak to outliers, results become less interpretable if features are highly correlated	Generalised Linear Model which extends linear regression for binary or multinomial classification tasks. Outputs probabilities of belonging to a class	Simple(r) classification tasks for which the data is mostly linearly separable	James et al. (2021)
Naïve Bayes	Simple and fast, works well with high‐dimensional data, performs well with independent features	Performs poorly with correlated features and/or if core assumptions are not held	Probabilistic family of algorithms which attempt to model the probability of an observation belonging to a target class, while maintaining the assumption that each feature contributes independently to such probability, irrespective of any possible inter‐feature correlations	Classification with independent features	Yang (2018)
KNN (k‐Nearest Neighbours)	Intuitive, no training phase, can model complex decision boundaries	Slower inference on larger datasets, performance degrades with high‐dimensional data (curse of dimensionality), weak to irrelevant features	Instance‐based lazy algorithm which classifies observations based on a majority vote of their k closest neighbours, determined via simple distance metrics (e.g. Euclidean, Manhattan, Hemming, etc.)	Classification tasks with smaller datasets and non‐linear, low‐dimensional decision boundary	James et al. (2021)
SVM (Support Vector Machines)	Effective in high‐dimensional spaces, robust to overfitting, especially in high‐dimensional data	Computationally intensive, difficult to tune (choice of kernel and regularisation), not efficient for large datasets	Type of algorithm which constructs an optimal p‐1 decision boundary hyperplane (p being the number of features of the input data) which maximises the margin between the closest observations of opposite classes, using data points that lie the closest to the hyperplane itself (support vectors) to determine the plane orientation and position	Particularly useful if data is non‐linearly separable while still presenting a clear margin of separation	James et al. (2021)
Gaussian Mixture Model	Can model complex distributions with multiple components, flexible in modelling uncertainty	Assumes data is normally distributed, may struggle with highly skewed or non‐Gaussian data, sensitive to initial conditions	Probabilistic unsupervised learning model, used for soft clustering tasks. It assumes that observed data points come from a mixture of multiple normal distributions, each with unknown mean and covariance	Clustering/anomaly detection	Jothilakshmi & Gudivada (2016)
Decision Trees	Easy to interpret, handles both numerical and categorical data	Prone to overfitting with especially with small datasets, sensitive to noisy data, limited ability to model complex relationships	Non‐parametric supervised learning model, which aims to represent a binary decision‐making process based on attributes (e.g. diagnoses). Internal nodes in the tree represent tests on attributes, branches represent attribute values and leaves represent the final predictions	Structured tabular data, diagnostics	James et al. (2021)
Random Forest	Reduces overfitting compared to decision trees, robust to noise, handles high‐dimensional data well, provides feature importance	Computationally expensive, can be slower to train and predict, difficult to interpret due to the ensemble nature	Ensemble model which averages the predictions of multiple decision trees together to produce stabler and more accurate results. Random forest classifiers split nodes based on the most informative feature among a random subset of features. Moreover, each tree is trained on a different bootstrapped subset of data to increase overall robustness	Structured tabular data, diagnostics	Breiman (2001)
XGBoost	Highly optimised, high performance, built‐in regularisation and cross validation, high flexibility	Can be computationally costly, may overfit if not properly regularised, sensible to outliers, lower interpretability compared to simpler models	Ensemble method in which decision trees are built sequentially to correct the mistakes of the previous iteration	Problems involving large, mixed‐type, structured data sets where accuracy is paramount	Chen and Guestrin (2016)
Gradient Boosting Machines	High performance, can handle complex data patterns, effective for imbalanced data	Computationally expensive, sequential architecture makes it overall slower and harder to parallelise	Ensemble method which builds multiple weak learners, iteratively reducing errors via gradient descent	Predictive analysis with complex decision boundaries	Natekin & Knoll (2013)
Artificial Neural Networks	Particularly adept at capturing highly complex trends in data, ability to learn new features automatically, highly scalable	High computational costs, data hungry, black box nature impacts interpretability, sensible to initialisation	Class of ML models which mimic the way biological neurons work. Each ANN consists of different layers of neurons, generally an input layer, one or more (deep‐learning neural networks) hidden layers and an output layer. Although different NN models exist, they typically share some strengths and drawbacks	/	Prieto et al. (2016)
MLPs (Multilayer Perceptrons)	Highly flexible, capable of modelling complex relationships, good for nonlinear problems	Prone to overfitting, require large amounts of data, difficult to interpret, sensitive to feature scaling and initialisation	A type of feed‐forward NN, where computations are performed sequentially without cycles (neurons cannot ‘feed’ data to themselves)	General purpose function approximations for non‐deep feature extraction tasks	Prieto et al. (2016)
CNNs (Convolutional Neural Networks)	Excellent for image and spatial data, captures hierarchical patterns, highly effective with large labelled datasets, can amortise computational costs via parameter sharing	Computationally intensive, requires large datasets, not ideal for non‐grid like data	A specialised type of Deep Neural Networks, particularly suited for computer vision tasks, and more generally for data that presents a grid‐like structure. They typically comprise three types of layers: convolutional, pooling and fully connected. While the first two perform feature extraction, the third maps the features into the output (e.g. classification)	Image processing, computer vision, object detection	Prieto et al. (2016)
RNNs (Recurrent Neural Networks)	Good for sequential data (time series, text), can capture temporal dependencies, suitable for tasks like language modelling	Difficult to train due to vanishing/exploding gradient problem, slow to train, struggles with long‐term dependencies (outside specific implementations)	Type of Deep Neural Networks typically used for sequential data. Their defining characteristic is their ‘memory’, meaning that, across layers, information from prior inputs is used to determine the current input and the overall output	Sequence modelling – short to medium length temporal dependencies	Prieto et al. (2016)

SVMs are supervised methods that can perform linear and non‐linear classifications, efficiently handling high‐dimensional data (Sen et al., 2020). This is achieved through the use of kernel functions that transform the input data into an implicit higher‐dimensional space in which linear separation is possible (Sen et al., 2020).

RNNs are purposefully designed to detect patterns in complex data sequences, especially time series data (Mao & Sejdić, 2022). This is possible because information is retained over time using common weights and activation functions (Mao & Sejdić 2022).

CNNs are based on convolutional layers that are able to detect hierarchical patterns within images, making them particularly effective at automatically detecting abnormalities in neuroimaging data (Rao & Sejdić 2022).

Ensemble methods are ML algorithms that aggregate the results of multiple models, often based on pseudorandom algorithms, resulting in improved prediction and generalisation performance. Notable ensemble methods algorithms include Random Forest and Gradient Boosting Machines (Stevens et al., 2023).

These systems have demonstrated their importance in new applications to clinical and neuropsychological data, ranging from the use of behavioural symptoms and other biomarkers to the use of clinical tests and, more generally, clinician observation.

## MACHINE LEARNING APPLICATIONS TO BEHAVIOURAL SYMPTOMS AND BIOMARKERS

Behavioural symptoms, such as voice and gaze, can provide essential but subtle clues to a patient's neuropsychological state, such as neurodegenerative disorders, aphasia and other disorders.

Similarly, biomarkers, that is, characteristics that can be objectively measured and evaluated as an index of normal or pathological processes (such as neuroimaging, biochemical or genetic data), can provide essential details to inform the clinical process.

All of these elements are not usually taken into account in neuropsychological assessments, which are mainly based on tests and clinical judgements. However, they provide an essential foundation for the neuropsychological evaluation crucial for differential analysis (see Table 2 for a summary).

**TABLE 2:** Accuracy, specificity, sensitivity, area under the curve (AUC) and F1 score in classifying neuropsychological diseases in ML applications.

Article	Classification algorithm	Groups	Sample size	Accuracy	Sensitivity	Specificity	AUC	F1‐score
Sharma & Giri (2014)	MLP, SVM, KNN	Parkinson's Disease (PD), Healthy Controls (HC)	23 PD, 8 HC	82.35% (MLP), 85. 29% (SVM), 82.35% (KNN)	84.61% (MLP), 100% (SVM), 84.61% (KNN)	75% (MLP), 37.5% (SVM), 75% (KNN)	NR	0.88%^a^ (MLP), 91.2%^a^ (SVM), 0.88%^a^ (KNN)
Tougui et al. (2022)	GBM	Parkinson's (P), Healthy Controls (HC)	Training set: 170 PD, 170 HC Hold‐out set: 42 PD, 42 HC	Training set: Lasso—71.76% ElasticNet—72.65% Hold‐out set: Lasso—71.43% ElasticNet—67.86%	Training set: Lasso—72.65% ElasticNet—70.29% Hold‐out set: Lasso—72.62% ElasticNet—67.86%	NR	NR	Training set: Lasso—72.01% ElasticNet—71.99% Hold‐out set: Lasso—71.76% ElasticNet—67.86%
Suppa et al. (2022)	SVM	Early‐stage Parkinson's (E‐SP), Mid‐advanced Parkinson's (M‐AP), Healthy Controls (HC)	57 E‐SP, 58 M‐AP, 108 HC	(Vowel/Sentence) 79.6%/77.3% (E‐SP) 93.5%/81.5% (M‐AP)	(Vowel/Sentence)87%/75.8% (E‐SP) 92.7%/82.7% (M‐AP)	(Vowel/Sentence) 77.4%/90.5% (E‐SP) 94.3%/80.4% (M‐AP)	(Vowel/Sentence) 0.90/0.88 (E‐SP) 0.98/0.90 (M‐AP)	NR
Fraser et al. (2013)	SVM, RF, NB	Semantic Variant PPA (svPPA), Non‐fluent Agrammatic PPA (nfaPPA), Healthy Controls (HC)	10 svPPA, 14 nfaPPA, 16 HC	82.13% (SVM), 84.85% (Random Forest), 77.48% (Naive Bayes)	NR	NR	NR	NR
Themistocleous et al. (2021)	DNN, SVM, RF, DT	Semantic Variant PPA (svPPA), Logopenic Variant PPA (lvPPA), Non‐fluent Agrammatic PPA (nfaPPA)	9 svPPA, 15 lvPPA, 19 nfaPPA	80% (DNN), 45%(SVM), 58% (RF), 57% (DT)	NR	NR	NR	NR
Tafuri et al. (2024)	R‐IML	Semantic Variant PPA (svPPA), Non‐fluent Agrammatic PPA (nfvPPA), Healthy Controls	31 svPPA, 25 nfvPPA, 53 HC	95% (svPPA vs. Controls), 77.1% (svPPA vs. nfvPPA) 93.7% (nfvPPA vs. Controls)	1.00 (svPPA vs. HC),1.00 (nfvPPA vs. HC), 0.67 (svPPA vs. nfvPPA)	0.90 (svPPA vs. HC), 0.88 (nfvPPA vs. HC), 0.88 (svPPA vs. nfvPPA)	0.99% (svPPA vs. HC), 0.98% (nfvPPA vs. HC), 1 (svPPA vs. nfvPPA)	0.95% (svPPA vs. HC), 0.93 (nfvPPA vs. HC), 0.93 (svPPA vs. nfvPPA)
Shu et al. (2021)	R‐iML	Mild Cognitive Impairment (MCI)	357 MCI	81.4% (training test), 80.7% (test)	82.2% (training test), 74.5% (test)	67.1% (training test), 73.8% (test)	NR	0.81% (12 months)
Yang et al. (2017)	SVM	Alzheimer's (A), Healthy Controls (HC), Mild Cognitive Impairment (MCI)	70 A, 150 HC, 98 MCI	97.7% (Alzheimer's vs. Controls), 87.8% (MCI vs. Controls)	99.2% (Alzheimer's vs. Controls), 86. 0% (MCI vs. Controls)	96.7% (Alzheimer's vs. Controls), 86.9% (MCI vs. Controls)	NR	NR
Haller et al. (2013)	SVM	Mild Cognitive Impairment (MCI), Single‐domain amnestic MCI (sd‐aMCI), Single‐domain frontal MCI (sd‐fMCI), Multi‐Domain Amnestic Mild Cognitive Impairment (md‐aMCI)	18 sd‐aMCI, 13 sd‐fMCI, 35 md‐aMCI	98.4% (md‐aMCI vs. sd‐fMCI), 97.7% (md‐aMCI vs. sd‐aMCI), 99.67% (sd‐fMCI vs. sd‐aMCI)	1% (md‐aMCI vs. sd‐fMCI), 1 (md‐aMCI vs. sd‐aMCI), 1% (sd‐fMCI vs. sd‐aMCI)	94.0% (md‐aMCI vs. sd‐fMCI), 93.1%^a^ (md‐aMCI vs. sd‐aMCI), 99.0%^a^ (sd‐fMCI vs. sd‐aMCI)	NR	97.1%^a^ (md‐aMCI vs. sd‐fMCI), 96.6%^a^ (md‐aMCI vs. sd‐aMCI), 99.5%^a^ (sd‐fMCI vs. sd‐aMCI)
Kristinsson et al. (2021)	SVR	Aphasia due to cerebral stroke	116	0% (fMRI—speech repetition), 61% (fractional anisotropy & lesion load), 53%–67% (integrated neuroimaging data)	NR	NR	NR	NR

*Note*: Article = the citation of the source.

Abbreviations: GBM, Gradient Boosting Machines; KNN, K‐nearest neighbours; MLP, multilayer perceptron; NB, Naïve Bayes; NR, not reported; RF, Random Forest; R‐iML, radiomics‐integrated ML; SVM, support vector machine.

^a^ When the article did not report Specificity, Sensitivity and/or F1, but there was the possibility to compute them, we applied the following formulas: Specificity = True Negatives/(True Negatives + False Positives); Sensitivity = True Positives/(True Positives + False Negatives); f1 = 2 × (sensitivity × recall) (sensitivity + recall), where recall = True Positives/(True Positives + False Negatives).

For example, neurodegenerative disorders affect eye movements because ocular fixation and gaze control involve a network that includes the cerebrum, brainstem and cerebellum, neural structures critically affected by neurodegenerative disorders (Anderson & MacAskill, 2013). For this reason, oculomotor signs are key to diagnosing neural degeneration (Anderson & MacAskill, 2013; Sekar et al., 2024) and can be a good candidate for automatic ML approaches. Indeed, ML algorithms applied to eye movements are able to find significant changes related to neurodegenerative symptoms (Przybyszewski et al., 2023).

Similarly, eye‐tracking data can be used to identify MCI compared to healthy control participants during a reading task with good performance (Groznik et al., 2021). Groznik et al. (2021) divided 115 participants into MCI or healthy controls, according to their performance at Addenbrooke‐ACER, Frontal Assessment Battery, Comprehensive Trail Making Test and emotional symptoms. Then, participants were required to read a text during eye‐tracking recording, considering several indexes computed from the fixations and saccades. The method showed a 73.1% classification accuracy.

The application of ML to eye‐tracking data during virtual reality scenarios can also be applied to identify stroke patients from healthy controls (Brouwer et al., 2022). For example, Brouwer et al. (2022) used SVM to eye‐tracking data during a task where they had to find either 3 or 7 items from a shopping list in a virtual supermarket environment. This methodology was able to identify stroke patients with 76% classification accuracy. The same combination of eye‐tracking and virtual reality scenarios was also applied to identify children with autistic spectrum disorder, showing good accuracy (86%) (Alcañiz et al., 2022).

Several neurological disorders can impact voice. From the most obvious aphasic disorders, voice can also be affected by MCI, Alzheimer's disease (Martínez‐Nicolás et al., 2021; Pulido et al., 2020), Parkinson's disease (Holmes et al., 2000), amyotrophic lateral sclerosis (Chiaramonte & Bonfiglio, 2020) and multiple sclerosis (Noffs et al., 2018). Interestingly, Sharma and Giri (2014), on a sample of 31 people (23 with Parkinson's disease and eight healthy controls, classified by diagnostic experts), collected voice data that were analysed and classified by MLP, KNN and SVM algorithms, showing the best accuracy for the SVM algorithm (85.29%). More recently, with a more representative sample of 424 participants (equally split into 212 with Parkinson's disease and 212 healthy controls), Tougui et al. (2022) applied Gradient Boosting Machines, reaching accuracy between 65% and 75%. Importantly, ML algorithms applied to voice data in Parkinson's disease are sensitive to the stage of the disorder and the L‐Dopa treatment. Indeed, Suppa et al. (2022) classified 115 patients affected by Parkinson's disease, divided into two subgroups: 57 early‐stage patients who never took L‐Dopa and 58 mid‐advanced‐stage patients who were chronically treated with L‐Dopa, against 108 healthy controls. Thirty‐one of the mid‐advanced‐stage patients were evaluated when OFF (after at least 12 h of L‐Dopa withdrawal) and ON therapy (1–2 h after the intake of L‐Dopa). SVM algorithms show an accuracy in classifying Parkinson's disease patients against healthy controls of 79.6% for vowels and 77.3% for sentences. Curiously, the accuracy in classifying early‐stage PD against healthy controls increases up to 81.5% for both vowels and sentences and in classifying mid‐advanced‐stage PD against controls at 93.5% for vowels and 81.5% for sentences. Notably, the accuracy in classifying between early‐stage and mid‐advanced‐stage PD patients was 88% for vowels and 92.5% for sentences and in classifying between mid‐advanced‐stage PD patients, ON and OFF L‐Dopa was 72.4% for vowels and 74.1% for sentences, suggesting the effectiveness of the system, that might apply even to phone interviews, reducing health costs.

Another interesting example is the use of speech data to identify variants in aphasic patients automatically. Primary progressive aphasia (PPA) is a neurodegenerative disease primarily affecting the language areas of the brain, which can be divided into three main variants: logopenic variant PPA (lvPPA), nonfluent agrammatic PPA (nfaPPA) and semantic variant PPA (svPPA) (Tippett, 2020). lvPPA is characterised by impairments during spontaneous speech in single‐word retrieval, naming and impaired repetition of phrases and sentences (Tippett, 2020). Individuals affected by nfaPPA show speech that is non‐fluent, requires considerable effort and is agrammatical. svPPA is different from the other two variants because it hinders semantic memory, leading to impaired object knowledge, anomia and single‐word comprehension deficits. These variants can be more broadly characterised as fluent (lvPPA and svPPA) and non‐fluent (nfaPPA) aphasia (Tippett, 2020).

Fraser et al. (2013) used SVM, NB and Random Forest algorithms to classify between svPPA and nfaPPA in a data set composed of speech samples from 10 svPPA, 14 nfaPPA and 16 healthy controls. Participants had to observe a figures‐only version of the “Cinderella” fairy tale without any text and, later, verbally explain the story without the book. Using the speech data and their transcription, SVM reached a mean accuracy of 82.13%, Random Forest reached a mean accuracy of 84.85% and NB reached a mean accuracy of 77.48%.

More recently, Themistocleous et al. (2021) used DNN to classify PPA patients in their variants, with a sample of 9 patients with svPPA, 15 with lvPPA and 19 with nfaPPA. The overall mean accuracy of the DNN was 80%, with 90% of nfaPPA and 95% of lvPPA correctly classified, while for svPPA, only 65% was correctly classified.

Interestingly, in a recent work, Metu et al. (2024) compared the performance of RNNs and CNNs with the Western Aphasia Battery‐Revised fluency scale (Kertesz, 2007) in identifying fluent and non‐fluent aphasic variants. The primary purpose of the work was to find an alternative to the fluency scale of the Western Aphasia Battery‐Revised, which is known to be characterised by low inter‐rater reliability and a lack of objectivity due to its reliance on subjective evaluations (Metu et al., 2024).

Speech data was collected from aphasic patients who were classified as fluent or non‐fluent by speech pathologists. Classifications based on the Western Aphasia Battery‐Revised fluency scale and speech data classified by RNNs and CNNs algorithms were then compared to the speech pathologist classifications. The results indicated only moderate agreement between the speech‐language pathologists and the Western Aphasia Battery‐Revised fluency scale, as well as between the pathologists and the neural network algorithms.

From these works, it seems that the application of ML to classify neurodegenerative disorders might lead to accuracies larger than 90%. However, this is true in particular when data samples are reduced, far from optimal, suggesting that these accuracies, more than reflecting the actual diagnostic value, might reflect overfitting of data. The implications are that the generalisability of such accuracies are doubtful and more work is needed to reach a true and consistent level of diagnostic accuracy desirable in clinical settings.

### Multimodal classification

It is worth noting that the use of multimodal data, including eye‐tracking, EEG, neuroimaging and behavioural indices, is promising in improving the accuracy of classification.

An interesting approach is the radiomics‐integrated ML approach. Radiomics refers to the use of high‐dimensional quantitative data extracted by neuroimaging, such as white matter texture, grey matter atrophy, lesion volume and other typologies of data (Mayerhoefer et al., 2020). In combination with clinical data and gene data, these data can be used in radiomics‐integrated ML approaches to establish a disease prediction model to improve prediction accuracy (Shu et al., 2021).

For example, in a recent work, Tafuri et al. (2024) applied radiomics‐integrated ML to white‐matter texture data, converted in 86 radiomics features from 56 patients with PPA (31 svPPA and 25 nfvPPA) and 53 healthy controls. These models demonstrated an accuracy of 95% in distinguishing svPPA patients from controls and 93.7% in distinguishing svPPA from nfvPPA. An accuracy of 93.7% was observed in differentiating nfvPPA patients from controls.

Shu et al. (2021) used a radiomics‐integrated ML model using white matter, grey matter and cerebrospinal fluid radiomics features, the level of apolipoprotein E4 (APOE4) and baseline Alzheimer's Disease Assessment Scale (Mohs, 1996), Clinical Dementia Rating scale (Morris, 1993) and Mini‐Mental State Examination scale (Folstein et al., 2014) to categorise patients between MCI, which after 4 years developed in Alzheimer's disease or not. Patients were 357, of whom 154 developed Alzheimer's disease within 4 years. The model was applied to a training set of 249 patients and to a test set of 108 patients. The accuracy of the model in the training set was 81.4%; while in the test set, it was 80.7%.

This approach was also used to differentiate between MCI subtypes: sd‐aMCI, showing isolated memory impairment; sd‐fMCI resulting in specific impairments of executive functions at early stages and md‐aMCI, characterised by a variety of symptoms, such as widespread cognitive dysfunctions that affect memory and also language, attention and/or visuospatial abilities (Forlenza et al., 2009). Haller et al. (2013) applied SVM to 66 MCI patients, composed of 18 sd‐aMCI, 13 sd‐fMCI and 35 md‐aMCI. White matter DTI data were used, and the SVM reached an accuracy of 98.4% in classifying md‐aMCI v. sd‐fMCI patients, 97.7% in classifying md‐aMCI and sd‐aMCI patients, and 99.67% in classifying sd‐fMCI and sd‐aMCI patients.

In another work (Yang et al., 2017), 70 patients with Alzheimer's Disease, 150 Healthy Controls, and 98 MCI patients were classified using an SVM algorithm on grey matter structure features, Mini‐Mental State Examination, Global Deterioration Scale (Solomon et al., 2014) and the Modified Hachinski Ischemic scale. The accuracy in classifying patients with Alzheimer's Disease and Healthy Controls was 97.7%, and the accuracy in classifying patients with MCI v. Healthy Control was 87.8%.

Kristinsson et al. (2021) used SVM to predict language function based on integrated neuroimaging data from 116 patients affected by aphasia caused by cerebral stroke. The language performance was estimated by using the Western Aphasia Battery (Kertesz, 2007) and the Western Aphasia Battery‐Revised (Kertesz, 2007), specifically focusing on fluency, spontaneous speech, naming, speech repetition, auditory comprehension and a score about overall aphasia severity. Neuroimaging data included task‐based functional magnetic resonance imaging (fMRI), diffusion‐based fractional anisotropy values, cerebral blood flow and lesion load and lesion volume data. The mean accuracy of the SVM algorithms based on the single neuroimaging data variable ranged between 0% (fMRI in predicting the speech repetition performance) to 61% (fractional anisotropy and lesion load, both predicting speech repetition performance). The mean accuracy of the integrated use of all the neuroimaging data in the SVM algorithm ranged from 53% to 67%, showing an improvement in the accuracy, even if it does not reach the minimum accuracy generally considered adequate (70%).

From these pieces of evidence, it appears that the use of neurophysiological data might be useful to classify neurodegenerative disorders. However, the accuracies of such approaches are extremely variable, with sample sizes that might not be considered adequate. Moreover, the use of neuroimaging and neurophysiological data poses scalability challenges in clinical settings, particularly when their predictive contribution is limited relative to cost. Also, in this case, further research is required.

### Theoretical advances

ML applications to biomarkers and behavioural symptoms seem to be confined to diagnostic tools, potentially useful for future health care in general and the neuropsychological field in particular, but with little impact on theoretical neuropsychological advances. However, this is not necessarily true; similar to what happened to well‐known and influential psychological theories, such as the personality theories (Ashton et al., 2004; McCrae & Costa Jr., 2008), data‐driven results can guide theoretical advances.

For example, Matias‐Guiu et al. (2019) applied ML techniques to FDG‐PET images acquired from PPA patients, identifying a total of five variants: Semantic variant, characterised by progressive loss of semantic knowledge in the context of otherwise well‐preserved language and cognitive abilities (corresponding to the already known svPPA); the nfaPPA variant was split into two specific variants: the nfaPPA type 1, associated with poorer performance in sentence repetition and irregular word reading, and the nfaPPA type 2, showing a higher degree of language apraxia; finally, the lvPPA was divided into two variants, the lvPPA type 1, performing poorly in action naming, and the lvPPA type 2, characterised by impairments in naming and sentence repetition. To the best of our knowledge, these subvariants are not included in PPA evaluations yet, but these might be informative in prognostic and rehabilitative terms.

A further example is the differentiation between people with autistic spectrum disorder in GeoPref and non‐GeoPref based on eye‐tracking data. Specifically, children with autistic spectrum disorder who observe videos of social or geometrical shapes tend to spend more time observing geometrical shapes and tend to manifest increased symptom severity than children with autistic spectrum disorder, but with a preference for observing social videos (Moore et al., 2018).

Thus, even if the application of ML has not improved neuropsychology yet from a theoretical point of view, this possibility is open and might be particularly viable using very large datasets, including different subvariants of the same pathology or more pathologies with specific characteristics (neuroanatomical or symptomatic) in common.

## SUPPORTING DIAGNOSTIC PROCESSES OF NEUROPSYCHOLOGICAL ASSESSMENT

ML is increasingly transforming neuropsychological assessment by enhancing diagnostic accuracy and streamlining the evaluation process.

### Enhance diagnostic accuracy

ML can analyse complex datasets from neuropsychological tests to identify patterns indicative of cognitive impairments. Several studies attempted to support diagnosis with ML. These studies are mostly based on the use of public datasets that allow for big (or relatively big) data, but there are also cases of studies run on newly acquired data, even in monocentric studies.

Particularly, the Alzheimer's disease continuum and Parkinson and Parkinsonisms have been targeted. We will focus on studies that used ML solely on clinical data of neuropsychological assessment.

Kang et al. (2019) investigated the application of deep learning algorithms to predict cognitive impairment using the Seoul Neuropsychological Screening Battery from the Clinical Research Center for Dementia in South Korea's dataset. The dataset comprises more than 14,000 formal neuropsychological assessments encompassing various cognitive domains. They aimed to distinguish between normal cognition, MCI and AD based on neuropsychological tests. An artificial neural network (ANN) model was trained using the TensorFlow framework to classify cognitive states. The model's performance was evaluated across 10 randomly selected datasets to ensure robustness. The artificial neural network model demonstrated high accuracy in distinguishing between NC, MCI and AD.

Adopting the same dataset, Simfukwe et al. (2024) utilised the SVM algorithm. The classifier was trained to differentiate between normal cognition, cognitive impairment and dementia based solely on test performance, achieving high classification accuracy, particularly in distinguishing normal cognition from dementia (97% accuracy).

While it may seem a relatively easy task to distinguish people with dementia from healthy individuals, ML can be used for more sophisticated differential diagnosis.

Gurevich et al. (2017) investigated the potential of ML in distinguishing AD from other neurocognitive disorders using exclusively neuropsychological test data. In their study, 158 individuals with MCI or dementia were classified as having AD or non‐AD based on cerebrospinal fluid biomarkers. Participants underwent a comprehensive assessment utilising a neuropsychological tests battery that included cognitive tests targeting AD‐typical deficits. The ML model trained on this dataset achieved an overall classification accuracy of 82%, with the highest accuracy (89%) in early‐stage patients.

Wang et al. (2022) instead investigated the application of ML to enhance the diagnosis of MCI and dementia in individuals presenting with normal Mini‐Mental State Examination (MMSE). The idea was that traditional cognitive screening tools such as the MMSE may fail to detect early cognitive decline, necessitating more sensitive diagnostic approaches. The study utilised 375 participants, categorised into cognitively unimpaired, MCI and dementia groups. Five algorithms (Logistic Regression, Decision Tree, SVM, XGBoost and RF) were trained on neuropsychological test battery scores to classify participants. Among these models, the RF algorithm demonstrated the highest diagnostic accuracy, achieving an area under the curve (AUC) of 0.89 for MCI detection and 0.84 for dementia classification.

Also, the domain of PD has been targeted. Reena Roy et al. (2024) explored the application of XGBoost, a powerful gradient‐boosting ML algorithm, for improving the diagnosis of PD using neuropsychological and clinical assessment data. The study aimed to enhance diagnostic accuracy while maintaining model interpretability through SHapley Additive Explanations (SHAP), an explainable AI framework that identifies the most influential features contributing to model predictions. XGBoost demonstrated superior classification performance, outperforming traditional ML methods in distinguishing PD patients from healthy individuals; SHAP analysis revealed that cognitive, motor and neuropsychological measures, such as executive function and processing speed, played a crucial role in predicting PD status.

Bougea et al. (2022) investigated the application of ML to distinguish between Dementia with Lewy Bodies and PD, again solely based on clinical and neuropsychological test scores. These two neurodegenerative disorders present overlapping cognitive and motor symptoms, making differential diagnosis challenging through traditional clinical methods alone. The study utilised multiple ML models, including Logistic Regression, K‐NN, SVM, NB and an ensemble model, to classify patients. Among the models tested, K‐NN demonstrated the highest classification accuracy (91.2%), distinguishing between Dementia with Lewy Bodies and PD.

These findings suggest that neuropsychological test patterns alone can effectively classify cognitive status, highlighting the potential of ML‐driven neuropsychological assessments in facilitating differential diagnosis of dementia or improving early dementia detection, even without reliance on neuroimaging or biomarker analysis and reducing diagnostic uncertainty in neurodegenerative diseases where symptom overlap complicates traditional assessments.

### Streamlining of the neuropsychological assessment

Another contribution of ML in neuropsychological assessment is to individuate the most important tests for classification. The analysis of which test matters the most to achieve a proper classification is called feature importance analysis and can be used to optimise neuropsychological batteries. The idea is to identify the most informative subset of cognitive tests to enhance classification accuracy while reducing the testing burden.

For example, Longo et al. (2024) explored the application of a Random Forest (RF) classifier to improve the neuropsychological assessment of mild cognitive impairment in PD. The study evaluated whether current diagnostic criteria comprehensively capture cognitive deficits in PD patients and whether incorporating social cognition assessments could enhance diagnostic accuracy. The algorithm was trained on 207 PD patients and tested on an independent sample of 68 PD patients. Four cognitive assessment batteries were created: two standard batteries (Levels I and II) and two alternative batteries (Levels I and II, incorporating a social cognition test). The results demonstrated that the alternative Level I (i.e., short) assessment had a diagnostic performance comparable to the standard Level II (i.e., long) assessment (AUC = 0.898 vs. 0.892). Moreover, the paper highlights the most predictive tests for distinguishing PD‐MCI from cognitively unimpaired PD patients, showing the importance of specific tests like the Trail Making Test B‐A, Ekman Emotion Recognition Test, Rey Auditory Verbal Learning Test–Immediate Recall.

Battista et al. (2017) explored the application of ML to enhance the efficiency and accuracy of neuropsychological assessments in classifying cognitive, behavioural and functional impairments in the Alzheimer's disease continuum. The study aimed to determine whether a reduced set of neuropsychological tests could effectively distinguish between varying levels of impairment, thereby streamlining the diagnostic process.

Utilising data from the Alzheimer's Disease Neuroimaging Initiative (ADNI) database, the researchers analysed 12 standard neuropsychological tests across participants categorised by the Clinical Dementia Rating scale into none, mild or severe impairment groups. The findings indicated that the Logical Memory test, specific components of the ADAS‐Cog, such as delayed and immediate memory recall, along with the FAQ's assessment of financial management abilities, are crucial predictors and suggest that a subset of tests is sufficient to achieve similar diagnostic accuracy.

Kleiman et al. (2021) also worked on the ADNI database to enhance the early detection of AD by identifying optimised sets of cognitive assessment features. Individuals were classified into three groups: cognitively normal, MCI and AD. Supervised feature selection methods were employed to identify cognitive assessment features most predictive of impairment, particularly at a Clinical Dementia Rating of 0.5 and above; they compared the efficacy of two‐class (impaired vs. non‐impaired) and three‐class (cognitively normal, MCI, AD) classification approaches. The optimised feature sets demonstrated improved sensitivity in detecting early‐stage cognitive impairment. The study found that certain combinations of cognitive assessments could effectively distinguish between different levels of impairment, suggesting the potential for more efficient and accurate screening protocols.

In a similar vein, Wang et al. (2022) identified six neuropsychological subtests as the most predictive features to distinguish MCI and dementia in individuals presenting with above‐threshold Mini‐Mental State Examination (MMSE), enabling the creation of an optimised neuropsychological test battery that reduced testing time while maintaining diagnostic accuracy.

Finally, Gupta and Kahali (2020) investigated the application of ML for the classification of cognitive impairment using an optimal combination of neuropsychological tests. The authors utilised multiple ML algorithms, including SVM and RF, to classify participants into healthy controls, MCI and AD groups based on neuropsychological test scores. Results showed that the optimised subset of tests improved classification accuracy without requiring the full test battery. The RF achieved the highest performance (96% accuracy), demonstrating that a carefully selected subset of cognitive assessments could effectively distinguish between healthy controls, MCI and AD.

ML‐driven approaches have the potential to refine cognitive assessment strategies in neurodegenerative disorders, ensuring diagnostic efficiency and enabling more accessible and less time‐consuming cognitive assessments in clinical and research settings. They also allow the individuation of critical but underexplored cognitive domains, such as social cognition. Results suggest that a focused subset of neuropsychological assessments can achieve comparable diagnostic accuracy to a more extensive test battery. Implementing such an optimised assessment approach could reduce the burden on patients and clinicians, facilitating earlier and more efficient detection of cognitive impairments.

## ADVANTAGES AND CHALLENGES OF MACHINE LEARNING IN NEUROPSYCHOLOGICAL ASSESSMENT

ML‐based approaches offer several advantages over traditional neuropsychological diagnostic methods, particularly in terms of objectivity, early detection and data efficiency. Unlike clinician‐dependent assessments, which may introduce variability in interpretation, ML models provide consistent and objective evaluations, reducing reliance on subjective judgement. This is particularly important in the detection of neurodegenerative diseases, where early identification of cognitive decline is crucial. ML models can recognise subtle cognitive changes before they manifest into more severe clinical symptoms, enabling timely intervention. Furthermore, these models are capable of processing large and complex datasets, integrating multiple neuropsychological test scores simultaneously to identify diagnostic patterns that might be overlooked in conventional assessments.

Despite these advantages, several challenges hinder the full integration of ML into routine neuropsychological practice. One major limitation is the need for large and diverse training datasets. ML models require extensive, well‐annotated neuropsychological data to ensure generalisability across different populations, which is often difficult to obtain. Additionally, many ML algorithms' lack of explainability and interpretability remains a barrier to clinical adoption. Clinicians require transparent models that provide clear rationales for decision‐making, allowing for greater trust and usability in medical settings. Another critical issue is external validation; while several ML studies demonstrate high accuracy in controlled research environments, their real‐world effectiveness needs to be independently validated across diverse clinical settings before widespread implementation can occur.

Another aspect characterising the use of ML in neuropsychological classification is that high accuracies are often associated with small samples, suggesting that we are facing overfitting more than actually adequate systems. Moreover, in ML applications, patients' data are generally compared to those of healthy individuals, which does not represent the clinical *dilemma* that actual neuropsychologists face. Clinicians need accurate tools for differential analysis. Thus, ML algorithms should be trained to classify patients with different pathologies. Although this might initially reduce the accuracy of such systems, it offers some benefits. For example, using neuroanatomical and neurophysiological patients' data, together with data coming from neuropsychological tests, might lead to identifying novel similarities between different pathologies, identifying new subvariants, and the use of explainable ML would allow us to understand or confirm which aspects are in common or different between the various pathologies. This might lead to unprecedented theoretical insights that will benefit neuropsychology.

An important, yet underexplored, aspect in the development of ML models for neuropsychological syndrome classification is the potential of Transfer Learning. Transfer Learning is an ML technique that improves performance on a target task by leveraging knowledge acquired from a related source task (Hosna et al., 2022). This approach is particularly advantageous when working with small datasets, as it allows the model to be initially trained on a larger, analogous dataset before being fine‐tuned on the more limited target data.

A notable example of Transfer Learning in action is multilingual BERT—a model pre‐trained on multiple languages, which has demonstrated significantly improved performance compared to models trained on a single language (Conneau et al., 2020). This is especially effective when models are pre‐trained on high‐resource languages and later fine‐tuned on low‐resource languages, allowing the model to generalise better even with limited training data (Boujkian, 2024).

This strategy could prove particularly valuable in neuropsychology, where high‐quality datasets are often small and costly to obtain. By training ML models on larger datasets from more prevalent or better‐understood conditions and then fine‐tuning them on rarer syndromes, it may be possible to improve classification accuracy and robustness.

Another promising insight from multilingual BERT is its ability to identify data‐driven universal grammatical relations across different languages (Chi et al., 2020). Similarly, a comparable ML approach in neuropsychology could uncover universal cognitive or lesion‐based patterns shared across different syndromes—an outcome that would be highly valuable both theoretically, by advancing our understanding of cognitive architecture, and practically, by improving diagnostic tools and treatment planning.

ML represents a significant advancement in neuropsychological diagnostics, offering enhanced accuracy, early detection capabilities and more objective assessment methodologies. As these models continue to evolve, their integration into clinical practice has the potential to redefine cognitive assessment paradigms, leading to improved outcomes for individuals with cognitive impairments. Future research should prioritise the optimisation of ML algorithms for real‐world applications, ensuring that these technologies are not only accurate and effective but also ethically and equitably implemented across diverse populations.

## CONCLUSIONS

ML represents a significant advancement in neuropsychological diagnostics, offering enhanced accuracy, early detection capabilities and more objective assessment methodologies. As these models continue to evolve, their integration into clinical practice has the potential to redefine cognitive assessment paradigms, leading to improved outcomes for individuals with cognitive impairments.

This narrative review has examined how ML methods are being applied to neuropsychological data—particularly cognitive test performance—to classify neurodegenerative disorders such as Alzheimer's disease, Parkinson's disease and primary progressive aphasia. Notably, ML models can support differential diagnosis, streamline cognitive evaluations and identify key features from high‐dimensional test batteries. When coupled with explainable AI techniques, these approaches have the potential to maintain clinical interpretability while enhancing diagnostic power.

However, the translation of ML models into clinical neuropsychology is not without challenges. Issues such as overfitting, limited sample sizes and lack of external validation remain central to the field's ongoing development. Moreover, the generalisability of high‐performing models across settings, languages and patient populations remains uncertain, underscoring the need for collaborative efforts and harmonised data standards. In this context, decentralised approaches like Swarm Learning and the possibility of leveraging Transfer Learning offer promising alternatives that preserve data privacy while facilitating cross‐institutional model training (Hosna et al., 2022; Warnat‐Herresthal et al., 2021).

It is also critical to situate ML within the broader landscape of AI. While this paper has focused on supervised learning algorithms applied to structured neuropsychological data, future developments may integrate additional branches of AI—such as LLMs, unsupervised learning and reinforcement learning—to address novel clinical and theoretical questions. Nonetheless, for ML to have meaningful clinical impact, collaboration between data scientists, neuropsychologists and clinicians is essential to ensure that algorithms reflect real‐world diagnostic needs and ethical standards.

By refining how we classify, interpret and monitor cognitive disorders, ML offers neuropsychology both a tool and a conceptual framework for navigating the complexity of brain‐behaviour relationships in the digital age. With thoughtful implementation and interdisciplinary collaboration, these technologies can enhance—not replace—the human expertise at the heart of neuropsychological care.

## AUTHOR CONTRIBUTIONS

**Michele Scandola:** Conceptualization; writing – original draft; writing – review and editing; supervision; resources. **Maria Esposito:** Data curation; writing – review and editing; visualization. **Riccardo Guidotti:** Writing – review and editing; visualization; data curation. **Daniele Romano:** Writing – review and editing; conceptualization; writing – original draft; supervision; resources.

## CONFLICT OF INTEREST STATEMENT

All the authors have no conflicts of interest to disclose.

## Supporting information


Data S1:

## Data Availability

Data sharing not applicable to this article as no datasets were generated or analysed during the current study.
