# Supplementary material for: How artificial intelligence is shaping neuropsychology: A focus on cognitive assessment of neurodegenerative disorders
Source: J Neuropsychol. 2025 Aug 9;20(1):256–75. doi: 10.1111/jnp.70009 (PMC12976813; doi:10.1111/jnp.70009)
Supplement: Supplementary file 1 — Data S1: [file JNP-20-256-s001.docx]

# Glossary

Below we provide a short list of common terms in machine learning which are relevant to the article.

**Accuracy:** simple model evaluation metric in classification. It is computed as the total number of observations correctly classified over the total number of observations.

**Artificial Intelligence (AI):** is a broad field of computer science focused on creating machines that can perform tasks that typically require human intelligence. This includes things like learning, problem-solving, understanding language, and recognizing patterns.

**Bagging:** short for bootstrap aggregating. It is an ensemble method in which the original data is divided into smaller subsets by bootstrapping. Then, each subset is used to train an individual weak learner sequentially. The results are then averaged (in regression) or voted on (in classification) to reach a final estimate.

**Bias/variance trade-off:** concept referring to the fact that models with low bias will have high variance and vice versa. Intuitively, if two variables have a quadratic true relationship, using a model which uses only a constant line passing through the mean to describe it would result in low variance but high bias. Conversely, a model describing the curve which passes through all the points would be extremely sensitive to new observations, resulting in low bias and high variance. The last case would also be a prime example of overfitting, whereas the addition of new data completely invalidates the model structure.

**Bias:** Bias is the tendency of a model to consistently make the same kinds of errors due to incorrect assumptions. For example, if the true form of the relationship in the data is quadratic, a linear model fit on this data will have high bias.

**Boosting:** ensemble method in which an initial weak learner is fit on the training data, then subsequent models are trained sequentially on a modified version of the dataset. Specifically, after each pass, the observations in the original dataset are assigned weights based on if the previous learner classified them correctly. This process continues until either the whole training set has been correctly classified or a set maximum number of models is reached.

**Bootstrapping:** in statistics, a process to quantify the accuracy of an estimator by simulating the procedure of obtaining new independent samples from the population. This is achieved by drawing samples from the available data, with replacement, with which to compute the estimate of interest. After repeating this process many times, the mean and standard error of the estimate are computed, indicating its accuracy compared to the real statistic.

**Classification:** in the domain of supervised learning, a problem for which the response vector of interest consists of ‘labels’, i.e. qualitative data. For example, predicting whether a subject has a certain disease based on the concentration of a particular substance in their blood.

**Cross validation:** general name for techniques used to estimate the test error rate in the absence of a large test set, by *holding out* a portion of the training observations. An example is the validation set approach, in which the available data is split into a training set and a validation set, from which the test set error is estimated.

**Curse of dimensionality:** general term for a case in which a model performs poorly in higher-dimensional feature spaces, due to them becoming increasingly sparse. For example, a model which assigns labels to observations based on their neighbors will have problems when the feature space becomes enlarged, because the distance between points will increase dramatically. In simple words, higher dimensions make things more complicated, especially for algorithms that need esponentially more data.

**Ensemble methods:** a procedure in which multiple ‘weak learners’, i.e. models with mild accuracy, high variance, etc. are combined to produce better, more stable measurements. Notable examples are bagging, boosting and random forest models for decision trees.

**Explainable AI:** it is a set of techniques and methods that allow humans to understand and interpret how AI models make their decisions. Instead of being "black boxes," XAI aims to make AI systems more transparent, enabling us to see why a particular prediction or outcome was reached.

**F1 score:** harmonic mean of precision and recall, used in conjunction with other metrics to determine the source of potential problems. For example, combining F1 score with false positives rate helps to mitigate false positive errors.

**Feature(s):** variables in a dataset used as input to produce the final model output (prediction, classification, etc.). Equivalent to *independent variables/predictors* in traditional statistics.

**Hyperparameter(s):** general term referring to parameters of the model which are set in advance, determining some aspects of how the model operates. Because their initial value is arbitrary, they typically must be ‘tuned’ to maximize model performance. This typically amounts to searching for the values maximize our performance metric of interest, e.g. accuracy, precision, etc

**Kernel function:** in the context of machine learning, a kernel function is a function that quantifies the similarity between pairs of data points. They are central to reducing computational times when operating on implicit higher dimensional spaces.

**K-fold cross validation:** common model evaluation technique whereby the dataset is divided into k *folds* of approximately equal size, then one of the folds is treated as the validation set and the others as the training. This process is repeated until all folds have been used as the validation. The model error is then obtained by averaging all the other errors.

**Large Language Models (LLMs):** are a type of machine learning model specifically designed to understand, generate, and process human language. They are "large" because they're trained on immense amounts of text data, allowing them to perform tasks like writing articles, answering questions, translating languages, and even generating creative text, all by predicting the next most probable word in a sequence.

**Machine Learning (ML)**: is a subset of AI where computers learn from data without being explicitly programmed. Instead of being given step-by-step instructions, ML models identify patterns and make predictions or decisions based on the data they've "seen."

**Out of bag error estimation:** it is a way to estimate how well a model will perform on unseen data—without needing a separate test set. In short, a single given weak learner model will make use of around two-thirds of the observations. The observations not used are called *out-of-bag*. Thus, to estimate a given observation, each model where the observation was *out-of-bag* is used, which produces around $\frac{B}{3}$ predictions for that observation, where $B$ is the number of bootstrap samples. By then averaging or taking a majority vote, one can produce a final prediction.

**Overfitting:** a case in which a mathematical model reproduces the data too closely, thus leading to poor generalization on new data, and overall lack of stability. *(see also bias/variance, underfitting)*

**Precision:** model evaluation metric in classification, used to evaluate ‘correctness’. It is computed as the ratio of true positives over the total number of positives predicted.

**Random forest:** ensemble method for decision trees where, much like bagging, each individual tree is trained only on a subset of observations. However here, each tree, at each split, is allowed to only choose a subset of predictors on which to perform the split. This strategy is employed to reduce the correlation between the individual weak learners, thus producing lower test error.

**Recall:** model evaluation metric in classification computed as the ratio of true positives over the total number of empirically positive cases.

**Regression:** in the domain of supervised learning, a problem for which the response vector of interest consists of continuous data. As the name suggests, many forms of linear regression fall in this category.^^[[1]](#footnote-1)^^

**Supervised learning:** concept referring to the situation in which a statistical learning model is trained on data with an associated response vector. In this sense, the model can be ‘fit’ to the response data. A classical example of a supervised learning sub-family of models is logistic regression.

**Swarm Learning (SL)**: is a decentralized machine learning method where many participants collaboratively train an AI model without sharing their raw data. Local models are trained on private data. However, in Swarm Learning, blockchain technology is often used to securely and transparently coordinate the sharing and aggregation of only the model insights (parameters/updates) among participants, eliminating the need for a central server.

**Training set:** The data used to train the model is aptly called the training set

**Test set:** in statistical learning, it is often the case that one wishes to build a model capable of generalizing well to new, unseen data. The data on which we wish to make predictions is the test set. They are unseen by the learning algorithm.

**Underfitting:** the opposite of overfitting, i.e. a case where a mathematical model does not capture the underlying structure of the data in a satisfactory way, owing to a lack of parameters.

**Unsupervised learning:** contrary to the supervised setting, a situation or problem for which a response vector is not available. In this case, the model learns patterns between predictors/observations without being able to refer to the empirical response variable. A popular problem in the unsupervised learning domain is *clustering*, whereby a researcher may be interested in uncovering whether observations form naturally distinct groups based on a set of predictors.

**Variance:** attribute of models referring to how much the model estimate would change when introducing new observations. For example, if one uses a very flexible model on a small dataset, the model might fit every tiny detail, including noise. This leads to high variance—its predictions will change a lot with even slight changes in the data.

1. It is important to note that the distinction between classification and regression is based on the type of problem at hand, as well as the type of output we wish to produce. While in classical statistics we may wish to use logistic regression to predict the probabilities of belonging to a category given a set of predictors, in statistical learning we instead wish to also label the observations using those probabilities. Thus, logistic regression can answer classification problems. [↑](#footnote-ref-1)
